# Inhibition of Exosome Release Alleviates Cognitive Impairment After Repetitive Mild Traumatic Brain Injury

**DOI:** 10.3389/fncel.2022.832140

**Published:** 2022-01-27

**Authors:** Tianpeng Hu, Zhaoli Han, Xiangyang Xiong, Meimei Li, Mengtian Guo, Zhenyu Yin, Dong Wang, Lu Cheng, Dai Li, Shishuang Zhang, Lu Wang, Jing Zhao, Qiang Liu, Fanglian Chen, Ping Lei

**Affiliations:** ^1^Department of Geriatrics, Tianjin Medical University General Hospital, Tianjin, China; ^2^Tianjin Neurological Institute, Tianjin Medical University General Hospital, Tianjin, China

**Keywords:** repetitive mild traumatic brain injury, chronic traumatic encephalopathy, exosome, microglia, cytokine, neuropathological protein, cognition, GW4869

## Abstract

**Background:**

Repetitive mild traumatic brain injury (rmTBI) is closely associated with chronic traumatic encephalopathy (CTE). Neuroinflammation and neuropathological protein accumulation are key links to CTE progression. Exosomes play important roles in neuroinflammation and neuropathological protein accumulation and spread. Here, we explored the role of brain-derived exosomes (BDEs) in mice with rmTBI and how the inhibition of BDE release contributes to neuroprotection.

**Methods:**

GW4869 was used to inhibit exosome release, and behavioural tests, PET/CT and western blotting were conducted to explore the impact of this inhibition from different perspectives. We further evaluated cytokine expression by Luminex and microglial activation by immunofluorescence in mice with rmTBI after exosome release inhibition.

**Results:**

Inhibition of BDE release reversed cognitive impairment in mice with rmTBI, enhanced glucose uptake and decreased neuropathological protein expression. Inhibition of BDE release also changed cytokine production trends and enhanced microglial proliferation.

**Conclusion:**

In this study, we found that BDEs are key factor in cognitive impairment in mice with rmTBI and that microglia are the main target of BDEs. Thus, inhibition of exosome release may be a new strategy for improving CTE prognoses.

## Introduction

Traumatic brain injury (TBI) is one of the leading causes of death and disability worldwide (Rubiano et al., 2015; Jiang et al., 2019). More than 50 million people suffer from TBI each year, and the annual cost of TBI is estimated to be over 500 billion dollars (Maas et al., 2017). TBI can be divided into three different categories based on injury severity: mild, moderate, and severe TBI. Among these categories, mild TBI is typically caused by non-penetrating head trauma and has various symptoms, including physical, cognitive, and behavioural symptoms as well as loss of consciousness (Blennow et al., 2016). Repetitive mild traumatic brain injury (rmTBI) has been shown to be closely associated with the development of chronic traumatic encephalopathy (CTE) in many studies (Smith et al., 2013; Wilson et al., 2017; Iverson et al., 2019). At present, the diagnosis of CTE still relies on autopsy, the lack of imaging and biomarkers for diagnosis limits the treatment of CTE (Turner et al., 2012). In order to explore the mechanism of CTE, preclinical trauma models have been used worldwide to conduct research on CTE. However, the rodent models used by various research groups are not the same in terms of many aspects such as injury interval, number of impacts and severity of injury (Turner et al., 2015). The impact acceleration (I/A) model, as a classic TBI damage model, was developed to stimulate mild, moderate, and severe TBI by using different weights and the velocity of impact to cause different degrees of damage (Yan et al., 2013; Hellewell et al., 2016). However, compared with rats, the damage degree of the weight-drop injury model in mice is relatively more difficult to control. Therefore, the controlled cortical impact (CCI) model is currently used to induce mild traumatic brain injury (mTBI) on mice (Ge et al., 2018). CTE is a distinctive tau protein-related neurodegenerative disease with unique characteristics including phosphorylated tau (p-TAU) accumulation in sulci and perivascular regions, microgliosis, and astrocytosis (DeKosky et al., 2013; Fesharaki-Zadeh, 2019; Smith et al., 2019). Studies have also shown that acetylated tau is highly correlated with the progression of CTE, and acetylation of tau will expose more phosphorylation sites, which will further cause the production of tau oligomers and neurofibrillary tangles (Lucke-Wold et al., 2014, 2017). Current studies have shown that neuroinflammatory reactions play important roles in the mechanism underlying CTE (Rubenstein et al., 2017). However, the mechanism is still unknown.

Microglia are innate immune cells in the brain and play an important role in the immunoinflammatory response (Gyoneva and Ransohoff, 2015). During the pathogenesis of CTE, the early phase of the inflammatory response triggered by mechanical damage is a neuroprotective response, but continuous inflammatory activation can cause secondary injury after months or even years (Corps et al., 2015). The initial injury in TBI will lead to an increase in the production of reactive oxygen species (ROS) and nitric oxide (NO), thereby triggering a state of oxidative stress. The state of oxidative stress will further directly activate inflammatory factors, thereby mediating the secondary injury after TBI(Abdul-Muneer et al., 2015; Ismail et al., 2020). Therefore, analysis and modulation of microglial proliferation and activation are very important for controlling the pathological development of CTE in immunoinflammatory reactions in the central nervous system (CNS).

Exosomes are small vesicles with diameters of ∼30 to ∼200 nm and are enriched in proteins, lipids, and nucleic acids, including mRNAs and miRNAs (Pegtel and Gould, 2019). The ability of exosomes to enrich and transfer cargos from donor to recipient cells makes them a vital part of cellular communication (O’Brien et al., 2020). Exosomes from different sources have specific impacts on recipient cells based on differences in the microenvironment (Goetzl et al., 2019; Mathieu et al., 2019). In our previous study, we found that microglia-derived exosomes could exert neuroprotective effects after rmTBI (Huang et al., 2018; Ge et al., 2020). However, the role of brain-derived exosomes (BDEs) in rmTBI is still unknown.

The neuroinflammatory reaction is one of the most important parts of the pathological process after rmTBI (Corps et al., 2015). The initial injury could lead to the secretion of cytokines after rmTBI, and subsequent injuries may cause a variety of changes in cytokine accumulation (Witcher et al., 2015; Jassam et al., 2017). Cytokines produced after rmTBI can influence the proliferation and activation of innate immune cells such as microglia and astrocytes. These cytokines could also trigger the recruitment and activation of peripheral immune cells (Morganti-Kossmann et al., 2019). One study showed that in a rat rmTBI model, the expression of IL-6 and IL-10 peaked at 2 weeks after injury and then decreased, which was accompanied by similar changes in microglial activation (Bai et al., 2017). Another study analysed cytokine production at 4 h after three repetitive mild closed-head injuries, and this study demonstrated a change in the expression of cytokines in the acute phase, including the upregulation of IL-15, MIP-1β, M-CSF, and IL-6 expression (Sankar et al., 2019). However, none of these studies revealed how cytokine levels change and how these changes are regulated in the long term after rmTBI. Therefore, this is important to clarify and may be a crucial target for CTE therapy.

## Materials and Methods

### Animals

C57BL/6J mice (8 weeks old, weighing 22–24 g) were purchased from the Chinese Academy of Military Science (Beijing, China). All the experimental procedures were performed in accordance with the NIH Guide for the Care and Use of Laboratory Animals under a protocol approved by the Tianjin Medical University Animal Care and Use Committee. The mice were housed and allowed to acclimate for 1 week before the experiments.

### Controlled Cortical Impact-Induced rmTBI Mouse Model

The rmTBI mouse model was established as we described previously (Ge et al., 2018). Briefly, the mice were anaesthetized with 4.6% isoflurane and then subjected to closed-head injury. A moulded acrylic cast was used to hold the mice in position. The mice were secured in a prone position on the acrylic cast with surgical tape across the shoulders. After shaving the head, a self-designed standard manufacturing concave metal disc was adhered to the skull immediately caudal to the bregma and used as a helmet. The impounder tip of the CCI device (model 6.3, American Instruments, Richmond, VA, United States) was then extended to its full impact distance, positioned on the centre of the disc surface, and reset to produce an impact. The impact head was charged at 5.0 m/s, and the depth of impact was 2.5 mm. After the impact, the mice were placed in a well-ventilated cage at 37°C until they regained consciousness. Four impacts were performed every 48 h to establish repetitive mild injury.

### GW4869 Treatment

GW4869, a neutral sphingomyelinase (n-Smase) inhibitor, purchased from SELLECK CHEMICAL, was administered via intraperitoneal (IP) injection to inhibit exosome secretion (Tabatadze et al., 2010; Iguchi et al., 2016). Mice were randomly divided into two groups: the rmTBI + Vehicle group and the rmTBI + GW4869 group. GW4869 was dissolved in dimethyl sulfoxide (DMSO) at 2.5 mg/ml and stored at –20°C. The working solution was freshly prepared at a final concentration of 1.25 mg/kg with 0.9% NaCl before use. Immediately after rmTBI, the mice received an IP injection of GW4869 or an equal volume of 0.9% NaCl once every two days.

### Isolation of Exosomes From Brains

The brain samples used for exosome isolation were harvested from the sham group and rmTBI group at 1 dpi. The animals were sacrificed by transcardiac perfusion with cold PBS. To acquire the desired brain samples, we detached the bilateral cerebral cortex and hippocampus and incubated them with 5 ml MACS Tissue Storage Solution. The samples were cut into small pieces and digested with 5 U/ml Papin (Worthington, OH, United States) for 30 min at 37°C. The samples were then centrifuged at 2,000 × *g* for 10 min at 4°C, and then, the supernatants were centrifuged at 10,000 × *g* for 30 min at 4°C to remove the cell debris. The supernatants were collected and filtered through a 0.22-μm filter to remove large particles. Then, the samples were ultracentrifuged at 100,000 × *g* for 70 min at 4°C to harvest the total brain-derived exosomes.

### Identification and Quantification of Exosomes

Exosomes were resuspended in 300 μl PBS and diluted 1:200 with PBS before analysis. The size distribution and concentration of the particles were measured and analysed by a Nano Particle Tracking and Zeta Potential Distribution Analyzer (Particle Metrix, Meerbusch, Germany).

### Morris Water Maze Test

The Morris water maze test was conducted on mice with rmTBI at 28–34 dpi. During the training phase (28–33 dpi), the mice were placed in a water pool (diameter of 105 cm) and allowed to swim freely. A platform was placed under the surface of water, and the mice were allowed to swim for up to 90 s to explore the whole space and find the platform. The mice were trained four times a day at 30-min intervals for six consecutive days (28–33 dpi). The mice were introduced to the maze in one of quadrants (northeast, northwest, southwest and southeast) for training, and the mice were introduced in one quadrant for the trial. The platform was fixed at the original site during the training phase and was removed on the test day (34 dpi). The mice were tested at 34 dpi and allowed to swim freely for 90 s. Then, the escape latency (time to reach the platform location) was recorded and calculated.

### Novel Object Recognition Test

The novel object recognition test was conducted on mice with rmTBI at 28 dpi. The mice were allowed to freely explore a 40-cm × 40-cm × 50-cm open field box for 10 min before the test. Then, two objects were placed in the box, and the mice were allowed to explore and become familiar with them during the familiarity phase. A stop watch was used to record the time the mice spent with each object until 20 s of total exploration or 10 min was reached. The test session was conducted 6 h later. In this session, one of the two objects was replaced with a novel object, and the mice were allowed to explore the two objects freely for 10 min. The time spent exploring each object was recorded, and the ratio of time spent with the novel object to the total exploration time was calculated.

### Positron Emission Tomography Computed Tomography Assessment of Glucose Metabolism With 18F-Fluoro-2-Deoxy-D-Glucose (FDG) and TAU Metabolism With 18F-S16-TAU

Mice were administered 5 MBq of 18F-fluoro-2-deoxy-D-glucose ([^18^F]FDG) via IP injection or [^18^F]S16-TAU via IV injection after anaesthesia. Thirty minutes later, the mice were anaesthetized with 2% isoflurane gas in an oxygen flow and positioned on the bed of a micro-PET/CT scanner (Novel Medical, Beijing, China). The CT scanning procedure was conducted as follows: tube voltage: 80 V; tube current: 0.5 mA; field of view (FOV): 70 mm; layer thickness: 0.18 mm. The PET scanning procedure was conducted as follows: FOV: 70 mm; matrix: 140 × 140; reconstruction agreement: PET-OSEM-Recon; number of iterations: 40.

### Western Blotting

SDS/PAGE and immunoblotting were performed at 1, 7, 14, and 28 days postinjury as we previously reported (Yin et al., 2020). An 8% SDS polyacrylamide gel was used to analyse APP, Tau-5, p-Tau, and acetylated-Tau (K686). GAPDH was used as the internal control. The details of the antibodies, including their sources, catalogue numbers and dilutions, are shown in Table 1. The ChemiDoc XRS + Imaging System (Bio–Rad, Hercules, CA, United States) was used for densitometry analysis. Measurement of the mean pixel density of each band was conducted by ImageJ software.

**TABLE 1:** Details of the Antibodies used.

Antibody	Brand	Sources	Catalogue Number	Dilution
Anti-APP Antibody	Cell Signaling Technology	Rabbit	#2452	1:1,000
Phospho-TAU Antibody	Cell Signaling Technology	Rabbit	#11834	1:1,000
Anti-TAU Antibody	Abcam	Mouse	Ab80579	1:1,000
GAPDH	Cell Signaling Technology	Rabbit	#2118	1:1,000
Iba1	Abcam	Goat	ab5076	1:500
Tau (Acetyl Lys686) Polyclonal Antibody	Abbkine	Rabbit	ABP57444	1:500
Phospho-Stat3 Antibdy	Cell Signaling Technology	Rabbit	#9145	1:100
Toll-like Receptor 4 Antibody	Absin	Rabbit		1:200

### Immunofluorescence

Brain sections were fixed in 4% PFA for 30 min at room temperature and washed with PBS for 5 min twice. Then, the brain sections were treated with 3% BSA for 30 min at room temperature to block non-specific staining. The sections were incubated for 16 h at 4°C with goat anti-Iba1 monoclonal antibodies (1:500; ab5076; Abcam, United Kingdom). After primary antibody incubation, the sections were rinsed with PBS and incubated with secondary antibodies for 1 h at room temperature. DAPI (104139; Abcam) was used to stain the nuclei. The details of the antibodies, including their sources, catalogue numbers and dilutions, are shown in Table 1. Immunostaining was observed and photo documented using a confocal microscope (Olympus, Heidelberg, Germany).

### Luminex Liquid Suspension Chip Detection

Luminex liquid suspension chip detection was performed by Wayen Biotechnologies (Shanghai, China). The Bio-Plex Pro Mouse Cytokines Grp I Panel 23-plex kit was used according to the manufacturer’s instructions. Briefly, brain tissues were harvested from the rmTBI + vehicle group and rmTBI + GW4869 group at 0, 1, 28, and 42 dpi. Brain homogenates were incubated in 96-well plates embedded with diluent microbeads for 30 min at room temperature and then incubated with detection antibodies at room temperature for 30 min after the samples were discarded. Finally, streptavidin-PE was added to each well and incubated for 10 min. The values were read using a Luminex 200 system (Luminex Corporation, Austin, TX, United States).

### Statistics Analysis

All the values are expressed as the mean ± SD. Statistical analysis was performed with GraphPad Prism 8.0 (GraphPad Software). Two-tailed unpaired Student’s *t* test was used to determine the significance of differences between two groups. One-way ANOVA followed by Tukey’s *post hoc* test or by two-way ANOVA with multiple comparisons were used for comparisons of multigroup data. Differences between means were considered statistically significant when *p* < 0.05. Animal weight was used for randomisation and group allocation. No animals were excluded from analysis.

## Results

### Quantity of Exosomes in Mouse Brains After rmTBI

**FIGURE 1:**
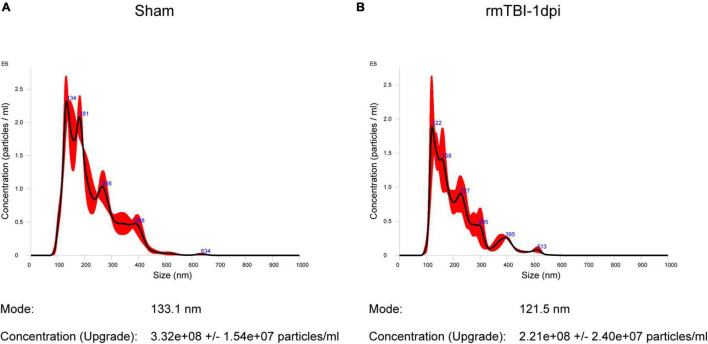
**(A,B)** Quantification of exosome concentrations using nanoparticle tracking analysis. **(A)** The mean diameter of the particles from the Sham group was 133.1 nm, and the concentration of the particles was 3.32 × 10^8^ ± 1.54 × 10^7^ particles/ml. **(B)** The mean diameter of the particles from the rmTBI group was 121.5 nm, and the concentration of the particles was 2.21 × 10^8^ ± 2.4 × 10^7^ particles/ml.

Exosomes, which are important tools for the transfer of materials between cells, play a crucial role during the process of CTE. We isolated BDEs and further quantified the concentrations of exosomes in mouse brains from the Sham group and rmTBI group at 1 dpi. The average diameter of the exosomes from the Sham group was 133.1 nm, and the concentration of the particles was 3.32 × 10^8^ particles/ml (Figure 1A). The average diameter of the exosomes from the rmTBI group was 121.5 nm, and the concentration of the particles was 2.21 × 10^8^ particles/ml (Figure 1B). The concentrations of the particles were not significantly different between the Sham and rmTBI groups.

### Inhibition of Exosome Release Improves Long-Term Cognition After rmTBI

**FIGURE 2:**
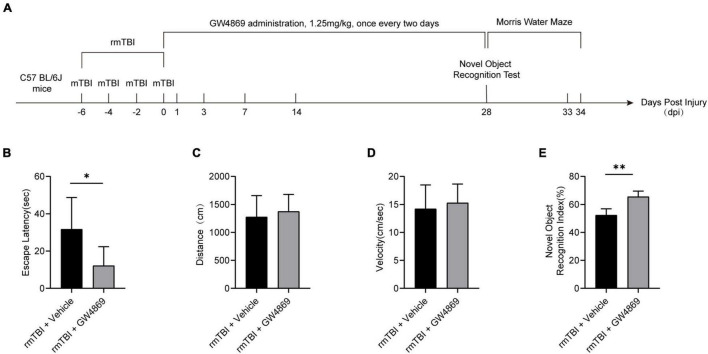
**(A)** Flow chart showing GW4869 administration and experimental design. C57BL/6J mice were subjected to rmTBI before GW4869 treatment. Immediately after rmTBI, the mice received GW4869 (1.25 mg/kg) or the same volume of 0.9% NaCl vehicle by intraperitoneal (IP) injection once every two days. A novel object recognition test was conducted at 28 dpi after rmTBI, and a Morris water maze test was conducted at 28–34 dpi after rmTBI. **(B–D)** Mice with rmTBI treated with GW4869 spent less time finding the platform during the Morris water maze test, but the swimming velocity and distance travelled were not significantly different between the mice in the rmTBI + Vehicle group and rmTBI + GW4869 group. Mean ± SEM. *n* = 10 mice per group. ^∗^*p* < 0.05. **(E)** Mice with rmTBI treated with GW4869 spent more time with the novel object than those treated with vehicle control. Mean ± SD. *n* = 8 mice per group. ^∗^*p* < 0.05, ^∗∗^ *p* < 0.01.

To explore the role of exosomes in rmTBI, we used GW4869 (a nMase inhibitor) to inhibit the secretion of exosomes after rmTBI and conducted cognitive tests with the Morris water maze and novel object recognition tests. The timeline of the experimental design is shown in Figure 2A. As shown in Figure 2B, in the Morris water maze experiment, the mice in the rmTBI + GW4869 group showed a great decrease in escape latency compared to those in the rmTBI + Vehicle group. However, the travel distance (Figure 2C) and velocity (Figure 2D) were not significantly different between the rmTBI + Vehicle group and the rmTBI + GW4869 group. In addition, the time spent with the novel object in the rmTBI + GW4869 group was much longer than that in the rmTBI + Vehicle group (Figure 2E).

### Inhibition of Exosome Release Enhances Glucose Metabolism and Attenuates Tau Levels in the Brain After rmTBI

**FIGURE 3:**
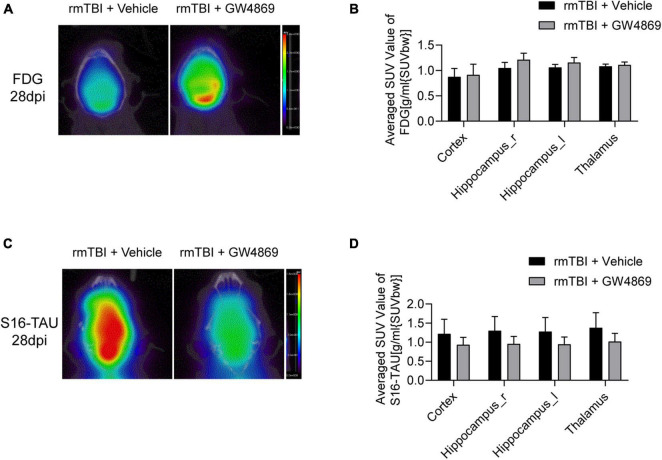
**(A)** Reconstructed [18F-FDG] PET/CT images of the mice in the rmTBI + Vehicle group and rmTBI + GW4869 group at 28 dpi. **(B)** Average SUV values of different areas of the brain, including the cortex, both sides of the hippocampus and the thalamus. **(B)** Bar graph showing that all the brain regions of the mice with rmTBI treated with GW4869 showed much higher glucose metabolism levels. Mean ± SD. *n* = 5 mice per group. **(C)** Reconstructed [S16-TAU] PET/CT images at 28 dpi after rmTBI and **(D)** bar graph showing that the TAU levels of the mice in the rmTBI + GW4869 group were much lower than those of the mice in the rmTBI + Vehicle group. Mean ± SD. *n* = 5 mice per group.

To further determine the impact of the inhibition of exosome secretion after rmTBI, we utilised small animal positron emission tomography computed tomography (PET/CT) to observe changes in cerebral imaging the mice. As shown in Figures 3A,B, the SUV values of [^18^F]FDG in the cortex, on both sides of the hippocampus and in the thalamus appeared to be much higher in the rmTBI + GW4869 group than in the rmTBI + Vehicle group 28 days after rmTBI. We also observed the cerebral TAU levels using a [^18^F]S16-TAU probe. Twenty-eight days after rmTBI, the SUV values of S16-TAU in the cortex, on both sides of the hippocampus and in the thalamus were substantially lower in the rmTBI + GW4869 group than in rmTBI + Vehicle group (Figures 3C,D).

### Inhibition of Exosome Release Reduces the Expression of Neuropathological Proteins

**FIGURE 4:**
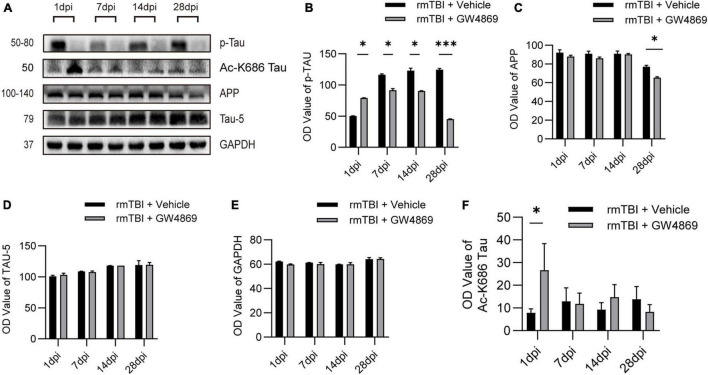
**(A)** Western blotting analysis of the expression of the neuropathological proteins APP, Aβ1-42, and p-Tau at 1, 7, 14, and 28 dpi in the brains of mice with rmTBI treated with GW4869 or Vehicle control. **(B)** Bar graph showing persistently lower levels of p-TAU in the rmTBI + GW4869 group versus the rmTBI + Vehicle group. The level of Aβ1-42 **(C)** decreased in the mice with rmTBI treated with GW4869 from 14 to 28 dpi after rmTBI. The level of APP **(D)** decreased in the mice with rmTBI treated with GW4869 at 28 dpi. **(E)** Bar graph showing that the expression of GAPDH remained unchanged. **(F)** Bar graph showing that compared with the rmTBI + Vehicle group, the acetylated-tau of the rmTBI + GW4869 group has a significant increase at 1dpi, but there is no significant change at 7, 14, and 28dpi. Mean ± SD. *n* = 5 mice per group. ^∗^*p* < 0.05, ^∗∗∗^*p* < 0.005.

To further study neuropathological changes that occur after the inhibition of exosome secretion, we used western blotting to verify changes in the expression of neuropathological proteins, such as p-TAU and APP. With long-term inhibition of exosome secretion we observed that the expression of p-TAU decreased in the rmTBI + GW4869 group compared with the rmTBI + Vehicle group (Figures 4A,B). However, the APP level declined only at 28 dpi after the inhibition of exosome secretion (Figures 4A,C). And the TAU-5 level remained unchanged after the inhibition of exosome secretion (Figures 4A,D). The expression of internal control GAPDH also remained unchanged (Figures 4A,E). We further tested the expression of acetylated-tau and found that the expression of acetylated-tau increased in the brain of rmTBI mice at 1dpi after exosome inhibition (Figures 4A,F). At the time point tested subsequently, inhibition of exosome release had no effect on the expression of acetylated-tau (Figures 4A,F).

### Inhibition of Exosome Release Alters the Secretion of Cytokines After rmTBI

**FIGURE 5:**
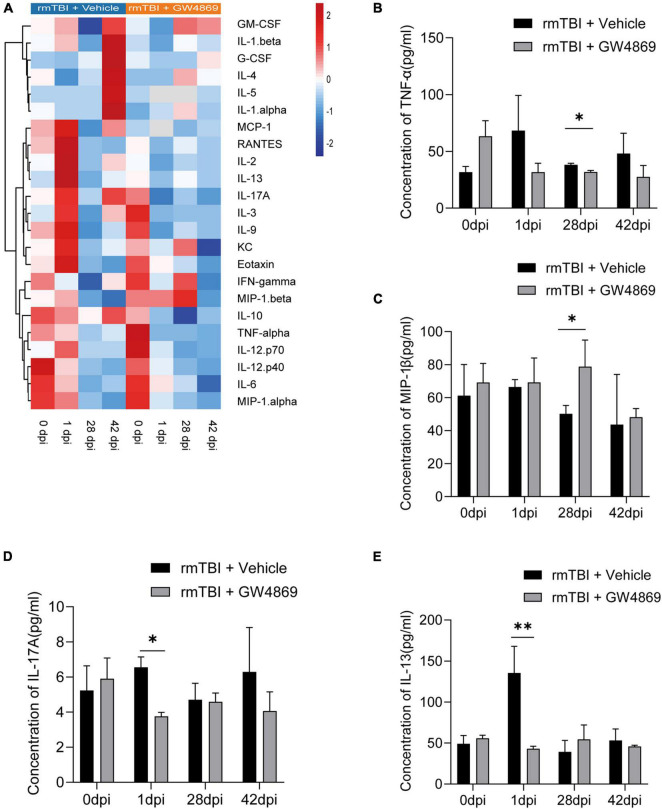
**(A)** Heatmap showing the cytokine expression profiles in the brains of mice with rmTBI treated with GW4869 or Vehicle control at 0, 1, 28, and 42 dpi after rmTBI. **(B–E)** Bar graphs showing different trends of cytokine expression in the of rmTBI + GW4869 group versus the rmTBI + Vehicle group. **(B)** Bar graph showing that the concentration of TNF-α decreased at 28 dpi after GW4869 treatment. **(C)** Bar graph showing that the concentration of MIP-1β increased at 28dpi after GW4869 treatment. **(D)** Bar graph showing that the concentration of IL-17A increased at 1dpi after GW4869 treatment. **(E)** Bar graph showing that the concentration of IL-13 decreased at 1dpi after GW4869 treatment. Mean ± SD. *n* = 3 mice per group. ^∗^*p* < 0.05, ^∗∗^ *p* < 0.01.

We further used Luminex to explore changes in cytokine production in mice with early- and late-stage rmTBI. Among all the cytokines we tested, we found multiple variation trends in mice after the inhibition of exosome secretion (Figure 5A). At 28dpi after rmTBI, TNF-α level decreased with GW4869 treatment (Figure 5B) and level of MIP-1α increased with GW4869 treatment (Figure 5C). However, some cytokines rapidly changed after GW4869 injection. The concentration of IL-17A and IL-13 showed a significant decline at 1dpi after GW4869 treatment (Figures 5D,E). These results suggested that it was feasible to regulate neuroinflammatory responses by inhibiting the secretion of exosomes to influence secretion of specific types of cytokines.

### Inhibition of Exosome Release Promotes the Activation of Microglia

**FIGURE 6:**
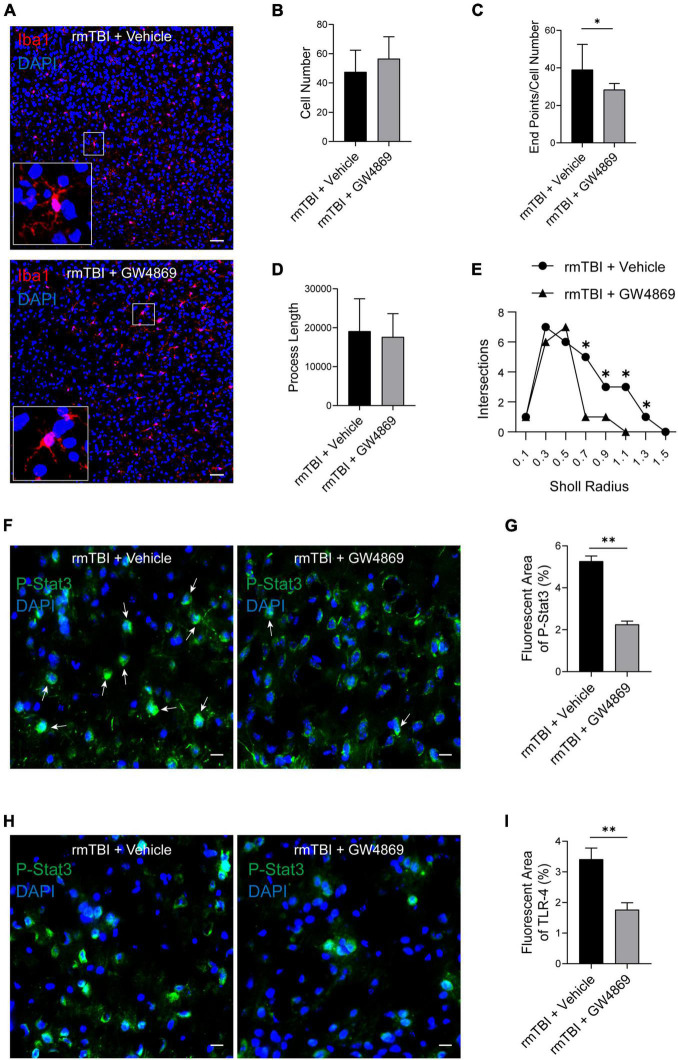
**(A)** Confocal images of Immunofluorescence stained microglia in the cerebral cortex of the mice from rmTBI + Vehicle group and rmTBI + GW4869 group at 1 dpi. Iba1 (red) + staining indicates microglia, and DAPI (blue) staining indicates nuclei. Scale bar, 50 μm. **(B)** Iba1 positive cells were counted and bar graph showing that there are no difference of Iba1 positive cell number between rmTBI + Vehicle group and rmTBI + GW4869 group. **(C)** Endpoints of microglia were counted by Image J, bar graph showing that the value of Endpoint/Cell Number in rmTBI + GW4869 group is lower than that in rmTBI + Vehicle group. **(D)** Total process lengths were measured by Image J and there are no difference of process length between rmTBI + Vehicle group and rmTBI + GW4869 group. **(E)** Sholl analysis was measured by Image J and line graph showing intersections of rmTBI + Vehicle group and rmTBI + GW4869 group. **(F)** Images of Immunofluorescence stained p-stat3(green) in the cerebral cortex of the mice from rmTBI + Vehicle group and rmTBI + GW4869 group at 1 dpi. White arrow showing p-stat3 and nuclear colocalisation. Scale bar 20 μm. **(G)** Bar graph showing that the immunofluorescence intensity of p-stat3 in rmTBI + GW4869 group is significantly lower than that in rmTBI + Vehicle group. **(H)** Images of Immunofluorescence stained toll-like receptor 4 (green) in the cerebral cortex of the mice from rmTBI + Vehicle group and rmTBI + GW4869 group at 1 dpi. Scale bar 20 μm. **(I)** Bar graph showing that the immunofluorescence intensity of TLR-4 in rmTBI + GW4869 group is significantly lower than that in rmTBI + Vehicle group. Mean ± SD. *n* = 6 mice per group. ^∗^*p* < 0.05, ^∗∗^ *p* < 0.01.

Microglia are innate immune cells in the brain and play important roles in endocrine and microenvironment regulation. Therefore, we used Iba1 as a marker of microglia to explore the impact of the inhibition of exosome secretion on microglia. As shown in Figures 6A,B, despite a slight elevation, there was no significant difference in the number of Iba1 + cells between the rmTBI + Vehicle group and the rmTBI + GW4869 group. To further explore the activation state of microglia with the inhibition of exosome secretion, we quantified microglia morphology by Image J. In the case of the same amount of Iba1 positive cells, the value of endpoints of microglia branches in rmTBI + GW4869 group was lower than that in rmTBI + Vehicle group (Figure 6C). The process lengths of microglia between rmTBI + Vehicle group and rmTBI + GW4869 group showed no significant difference (Figure 6D). And we also conducted sholl analysis with ImageJ and found that the number of intersections of different radii in rmTBI + GW4869 group was much lower than that in rmTBI + Vehicle group (Figure 6E). Less endpoints and intersections in rmTBI + GW4869 group indicated the activation of microglia, which means the inhibition of exosome release could enhance the activation of microglia. We further tested two common inflammatory response markers p-stat3 and toll-like receptor 4 (TLR-4). Through the method of immunofluorescence staining, we found that inhibiting the release of exosomes can inhibit the expression of p-stat3 on the one hand, and on the other hand, it can also significantly inhibit the entry of p-stat3 into the nucleus (Figures 6F,G). The TLR-4 immunofluorescence staining results also showed that inhibiting the release of exosomes can also significantly inhibit the activation of TLR-4 in the brain of rmTBI mice (Figures 6H,I).

## Discussion

Studies have shown that exosomes from different origins have different impacts on the prognosis of rmTBI (Huang et al., 2018; Yin et al., 2020). However, the role of exosomes in the pathological process of rmTBI remains unknown. In this study, we used multiple methods to determine the role of exosomes in rmTBI. First, we measured the exosome quantity of mice with rmTBI and found that there were no significant differences in particle numbers in mouse brains between the Sham group and rmTBI group; this finding suggested that the cargo carried by exosomes, but not the exosomes themselves, function during the process of rmTBI. Therefore, we used GW4869 (a nSmase inhibitor) to inhibit the secretion of exosomes in order to explore the effect of BDEs on mice with rmTBI. PET/CT imaging of mice with rmTBI showed that one injection of GW4869 slightly enhanced the cerebral extraction of glucose in mice with rmTBI (data not shown), which suggested that the inhibition of exosome secretion could improve the metabolism of mice with rmTBI. After 28 days of GW4869 administration, PET/CT imaging showed a more significant enhancement of glucose extraction in the cerebral tissue of mice with rmTBI. The results of behavioural tests, including the Morris water maze and novel object recognition tests, indicated that inhibiting the secretion of exosomes could markedly improve the cognition of mice with rmTBI. Western blotting analysis also showed that the inhibition of exosomes reduced the expression of neuropathological proteins, including p-TAU and APP. These results indicate that exosomes are key players in the pathological process of rmTBI and that the inhibition of exosome secretion could benefit the prognosis of rmTBI and CTE. Studies have suggested that the acetylation of tau may occur earlier than the phosphorylation of tau, and the acetylation of tau will expose more phosphorylation site(B. Lucke-Wold et al., 2017). Our results show that the inhibition of exosome secretion after rmTBI only affects the expression of acetylated tau in the early stage, but has no effect in the later stage of the injury. This result may be due to the acetylation sites we detected. It may be necessary to detect more tau acetylation sites to explore the relationship between the inhibition of exosome release and the decreased expression of p-tau. To further explore the function of exosomes, Luminex was conducted to evaluate the expression of cytokines in the brains of mice with rmTBI at the early and late stages, and we found some novel phenomena. GW4869 treatment resulted in opposite trends in the expression of cytokines such as IL-1α and IL-9 compared with the control treatment. Moreover, the expression of some cytokines did not differ between the rmTBI + Vehicle group and the rmTBI + GW4869 group. The changes that occurred after GW4869 administration were considered to be the result of the secretion of different cytokines. These results also give us a new way of regulating the secretion of specific kinds of cytokines and controlling the neuroinflammation process during rmTBI and CTE. More than 300 kinds of cytokines have currently been identified and are difficult to categorise (Becher et al., 2017). We tested only a small portion of cytokines, but the response of cytokine production to the inhibition of exosome secretion is still an interesting discovery, and this finding will help us to better understand other the responses of cytokines during the processes of rmTBI and CTE. On this basis, we further observed the proliferation and activation of microglia in mice with rmTBI; we observed a slight increase in the proliferation and great activation of microglia after GW4869 administration at 1 day postinjury, which suggests that BDEs could be key for regulating the proliferation and activation of microglia. These results suggested that during the neuropathological process of rmTBI, BDEs could target microglia and influence the activation of microglia to regulate glucose metabolism. We further tested the expression of p-stat3 and TLR-4, and we found that inhibiting the secretion of exosomes can significantly inhibit the expression of p-stat3 and TLR-4, and inhibiting the secretion of exosomes can also inhibit the entry of p-stat3 into the nucleus. But this change of p-stat3 and tlr-4 did not co-localise with microglia. We speculated that the changes caused by inhibiting exosome secretion may occur more in neurons, so as to improve the prognosis of neurological function in rmTBI mice. The neuroinflammatory response mediated by microglia plays a key role in central nervous system diseases. It has been reported that indomethacin can increase the number of neuroblasts by inhibiting the activation of microglia after focal ischaemia, thereby exerting a neuroprotective effect (Lopes et al., 2016). And minocycline can also reduce white matter damage by inhibiting the activation of microglia (Guimaraes et al., 2010), or play a transient neuroprotective effect by inhibiting the activation of microglia (Bye et al., 2007). Therefore, regulating the activation state of microglia is very important in the treatment of central nervous system diseases. The effect of exosomes also suggests that interventions targetting exosomes at different stages of disease may have different effects. These findings may provide a new direction for improving the prognosis of disease by treating rmTBI and CTE with therapies that target exosomes in the future.

## Data Availability Statement

The original contributions presented in the study are included in the article/supplementary material, further inquiries can be directed to the corresponding author/s.

## Ethics Statement

The animal study was reviewed and approved by Tianjin Medical University Animal Care and Use Committee.

## Author Contributions

PL and ZH were responsible for study design. TH developed the methodology. TH, ZH, XX, ML, MG, ZY, DW, LC, DL, SZ, LW, and JZ carried out the experiments. QL and FC provided technical support. TH and ZH interpreted the results, performed data analysis, prepared the figures and tables, and wrote the manuscript. PL supervised the study. All authors read and approved the final manuscript.

## Conflict of Interest

The authors declare that the research was conducted in the absence of any commercial or financial relationships that could be construed as a potential conflict of interest.

## Publisher’s Note

All claims expressed in this article are solely those of the authors and do not necessarily represent those of their affiliated organizations, or those of the publisher, the editors and the reviewers. Any product that may be evaluated in this article, or claim that may be made by its manufacturer, is not guaranteed or endorsed by the publisher.
